# Phenolic compounds in ectomycorrhizal interaction of lignin modified silver birch

**DOI:** 10.1186/1471-2229-9-124

**Published:** 2009-09-29

**Authors:** Suvi Sutela, Karoliina Niemi, Jaanika Edesi, Tapio Laakso, Pekka Saranpää, Jaana Vuosku, Riina Mäkelä, Heidi Tiimonen, Vincent L Chiang, Janne Koskimäki, Marja Suorsa, Riitta Julkunen-Tiitto, Hely Häggman

**Affiliations:** 1 Department of Biology, University of Oulu, PO Box 3000, 90014 Oulu, Finland; 2 Department of Applied Biology, University of Helsinki, PO Box 27, 00014 Helsinki, Finland; 3 Finnish Forest Research Institute, Vantaa Research Unit, Jokiniemenkuja 1, 01301 Vantaa, Finland; 4 Finnish Forest Research Institute, Punkaharju Research Unit, Finlandiantie 18, 58450 Punkaharju, Finland; 5 Forest Biotechnology Research Group, Department of Forestry and Environmental Resources, College of Natural Resources, North Carolina State University, Campus Box 7247, 2500, Partners II Building, Raleigh, NC 27695-7247, USA; 6 Department of Biology, University of Joensuu, PO Box 111, 80101 Joensuu, Finland

## Abstract

**Background:**

The monolignol biosynthetic pathway interconnects with the biosynthesis of other secondary phenolic metabolites, such as cinnamic acid derivatives, flavonoids and condensed tannins. The objective of this study is to evaluate whether genetic modification of the monolignol pathway in silver birch (*Betula pendula *Roth.) would alter the metabolism of these phenolic compounds and how such alterations, if exist, would affect the ectomycorrhizal symbiosis.

**Results:**

Silver birch lines expressing quaking aspen (*Populus tremuloides *L.) caffeate/5-hydroxyferulate *O*-methyltransferase (*PtCOMT*) under the 35S cauliflower mosaic virus (CaMV) promoter showed a reduction in the relative expression of a putative silver birch *COMT *(*BpCOMT*) gene and, consequently, a decrease in the lignin syringyl/guaiacyl composition ratio. Alterations were also detected in concentrations of certain phenolic compounds. All PtCOMT silver birch lines produced normal ectomycorrhizas with the ectomycorrhizal fungus *Paxillus involutus *(Batsch: Fr.), and the formation of symbiosis enhanced the growth of the transgenic plants.

**Conclusion:**

The down-regulation of *BpCOMT *in the 35S-PtCOMT lines caused a reduction in the syringyl/guaiacyl ratio of lignin, but no significant effect was seen in the composition or quantity of phenolic compounds that would have been caused by the expression of *PtCOMT *under the 35S or UbB1 promoter. Moreover, the detected alterations in the composition of lignin and secondary phenolic compounds had no effect on the interaction between silver birch and *P. involutus*.

## Background

The phenylpropanoid pathway gives rise to a variety of compounds that are used in the biosynthesis of cinnamic acid derivatives, lignin, flavonoids and condensed tannins. These phenolic compounds form a diverse group of secondary metabolites, exhibiting numerous biological functions in plants. In the *Betula *species, the phenolic compound concentrations vary according to the development phase of the plant [1,2], clone [2-4] or plant part [5] and to different environmental factors [2,4,5]. Moreover, tannins and specific flavonoids have been shown to play a role in defence against herbivory [6] and protection against UVB radiation [1,7-9]. In addition to the phenolic compound profiles of different *Betula *species, the general outline of the phenylpropanoid pathway of the species is also well known [4].

The secondary cell wall is essential for the conduction of water and the structural integrity of vascular plants as well as for defence against insect herbivores and pathogens. The secondary cell wall is composed of multiple layers of cellulose microfibrils embedded in a matrix of hemicellulose, lignin and pectin. Lignin, probably the most studied phenolic compound, is composed of monomers derived from the monolignol biosynthetic pathway [10]. In hardwoods, coniferyl, sinapyl and *p-*coumaryl alcohol are the main lignin monomers, giving rise to guaiacyl (G), syringyl (S) and *p-*hydroxyphenyl (H) phenylpropanoid units, respectively, when polymerized to the lignin molecule. These hydroxycinnamyl alcohols differ in their degree of methylation and, consequently, form varying linkage types in the lignin, determining the solubility of the polymer. In sinapyl alcohol, the C-5 position of the aromatic ring is methylated, which prevents the formation of strong linkage types that are typical for G units. Angiosperm lignin consists mainly of G and S monomers and is more easily delignified than the G unit rich gymnosperm lignin. The monolignol biosynthetic pathway is still under debate, partly because the enzymes involved in the pathway are multifunctional and exhibit broad substrate specificity, at least *in vitro*, making several alternative reaction orders possible. The most updated view of the angiosperm monolignol biosynthetic pathway is presented by Li et al. [11], Do et al. [12] and Vanholme et al. [13].

The caffeate/5-hydroxyferulate *O*-methyltransferase (COMT) (EC 2.1.1.68), also known as 5-hydroxyconiferyl aldehyde *O*-methyltransferase (AldOMT) [14] catalyses the methylation of the C-5 position of angiosperms' S precursors. COMT belongs to the plant Class II *O*-methyltransferases (OMTs) together with enzymes that methylate numerous phenolic compounds, such as phenylpropenes and flavonols [15,16]. Initially COMT was shown to use caffeic acid and 5-hydroxyferulate as substrates [17,18], but further studies demonstrated that COMT is especially involved in the biosynthesis of S lignin [19-22] and, furthermore, that the methylation occurs at 5-hydroxyconiferaldehyde and (or) 5-hydroxyconiferyl alcohol as shown with various angiosperm species [14,23-26]. However, the substrate preferences of COMT may vary between species being, for instance, relatively board in alfalfa (*Medicago sativa *L.) [24] and wheat (*Triticum aestivum *L.) [26]. Some of the enzymes having COMT activity are probably bifunctional as in the case of *Arabidopsis thaliana *OMT (At5g54160) which is involved in both lignin and flavonoid biosynthesis [12,27,28].

Silver birch (*Betula pendula *Roth) is one of the key species in boreal forest ecosystems and, in addition, economically the most important deciduous tree species in Nordic countries. In Finland, based on the national forest inventory performed during years 2004 through 2007 approximately 16% of growing stock was birch (363 mill. m^3^) [29]. The birch roundwood is used as a raw material in the chemical pulp industry but also in plywood production. Moreover, birch is an important source of energywood: in 2007 wood-based fuels covered one fifth (295PJ) of the total energy consumption in Finland [30].

Boreal forest trees live in a mutualistic association with ectomycorrhizal (ECM) fungi, which enables growth in the nutrient-poor, acidic soils. The formation of ECM symbiosis causes changes in the transcription levels of both partners [31-34], resulting in morphological and physiological alterations. The proliferation of root hairs is inhibited and the epidermal cells of feeder roots in angiosperms elongate radially as the fungus penetrates into the intercellular space of the epidermis. The fungal hyphae that cover feeder roots are also a source of an external hyphal net. These distinctive alterations in the symbiotic partners ensure the effective exchange of water and nutrients from the fungal partner to the carbohydrates of the plant [35]. ECM formation has also been observed to alter the expression levels of genes involved in the phenylpropanoid pathway [32,33,36] and the concentrations of phenolic compounds [37-43]. However, the results have been rather inconsistent.

Transgenic plants have great potential for future agriculture, silviculture and biofuel production. Increasing the pest and disease resistance of plants as well as improving wood quality and enhancing wood production have been the targets of both conventional breeding and genetic engineering. From an industrial point of view, lignin quality and content are of particular interest. The removal of lignin in chemical pulping is a costly process which could be facilitated with more soluble lignin and lower lignin content [44]. A reduction in lignin content would also be beneficial for the production of bioethanol [45]. Other processes related to the production of bioethanol could also be enhanced by modifications in the cell wall chemistry, as reviewed by Sticklen [46]. Lignin modifications using various gene constructs that are associated with the monolignol biosynthetic route have been conducted successfully on angiosperm tree species (reviewed in [10,11,13]).

Changes in the primary as well as in the secondary metabolism of organisms are triggered by a variety of stimuli, such as changes in the developmental phase or environmental factors. Therefore, the pleiotropic or non-target effects of transgenes should also be studied in diverse environmental conditions. So far, only minor changes have been found in interactions between lignin modified trees and herbivores or soil microfauna [47-53]. Recent studies investigating possible changes in the secondary metabolism that are caused by genetic transformations have mostly been conducted on herbaceous species [54-59] and without the involvement of ecological interactions. In the present study, we analyzed the phenolic compounds and lignin characteristics of PtCOMT silver birch lines (*Betula pendula *Roth.) in interaction with the ECM fungus *Paxillus involutus *(Batsch: Fr.) in order to determine the impact of the symbiosis on the phenylpropanoid route derived compounds and to detect possible unintended effects of transgene expression.

## Results

### Expression of *PtCOMT *and *BpCOMT *in roots

**Figure 1:**
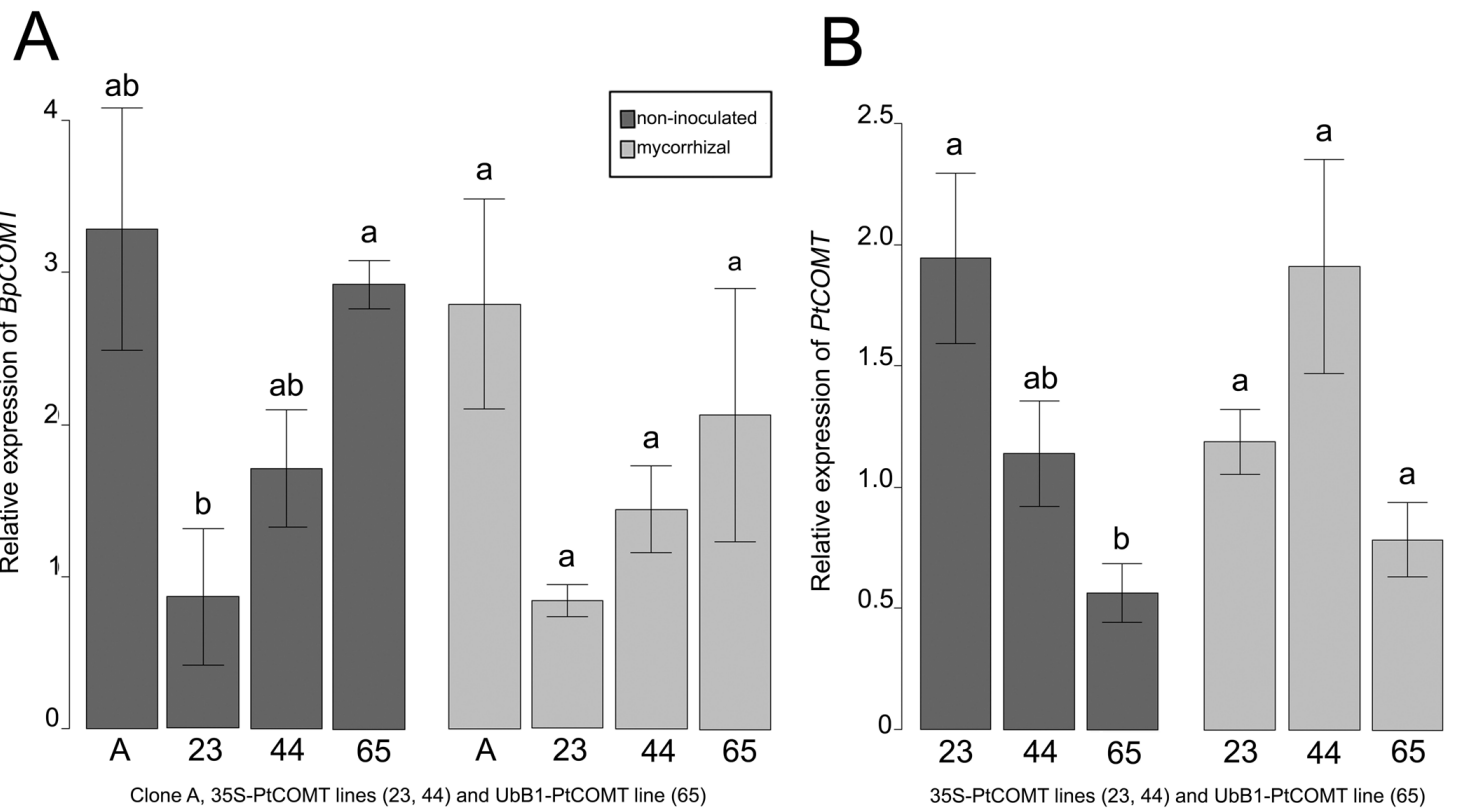
**RT-PCR results of *BpCOMT *and *PtCOMT *in silver birch roots**. Relative expression of the endogenous putative caffeate/5-hydroxyferulate *O*-methyltransferase of silver birch (*BpCOMT*) (A) and the heterologous *PtCOMT *gene (B) normalized using *atub *and putative *BpPP2A *as reference genes in the non-inoculated and mycorrhizal roots of clone A and PtCOMT-modified lines 23, 44 and 65. Values are means ± standard error. Different letters above the columns denote significant (*P *< 0.05) difference between the PtCOMT lines and clone A within the treatments according to the two-sample t-test or the Wilcoxon rank sum test with the Bonferroni correction. Number of replicates 3-5.

The open reading frame of putative *COMT *(*BpCOMT*) and partial sequence (1536 bp) of *PP2A *(*BpPP2A*) of silver birch were sequenced. The putative *BpCOMT *was 72% identical to *PtCOMT *[EMBL: X62096] at the nucleotide level and 87% identical at the amino acid level (Additional file 1) and showed highest similarity to the castor bean (*Ricinus communis*) COMT [GenBank: EEF36570] (90%) and almond (*Prunus dulcis*) COMT [EMBL: CAA58218] (88%). The putative *BpPP2A *showed 91% similarity with *Medicago sativa subsp*. x *varia *[GenBank: AAG29593] and 90% similarity with *A. thaliana *[GenBank: NP_172790] PP2A at the amino acid level (Additional file 2). The expression of *PtCOMT *and the putative *BpCOMT *was studied from the non-inoculated and mycorrhizal roots of silver birch (Figure 1A, B). The relative expression of the putative *BpCOMT *was similar in both non-inoculated and mycorrhizal roots: 35S-PtCOMT lines 23 and 44 had lower average levels of *BpCOMT *transcripts than UbB1-PtCOMT line 65 and clone A (Figure 1A). However, significant differences (*P *< 0.05) in the relative expression of *BpCOMT *were only observed in the non-inoculated roots between UbB1-PtCOMT line 65 and 35S-PtCOMT line 23. The relative expression of *PtCOMT *was significantly (*P *< 0.05) higher in the non-inoculated roots of 35S-PtCOMT line 23 than in line 65, where the transgene was driven by the UbB1 promoter (Figure 1B). In mycorrhizal roots, the relative expression levels of *PtCOMT *between lines were comparable to those of non-inoculated roots.

### Lignin distribution and composition

**Figure 2:**
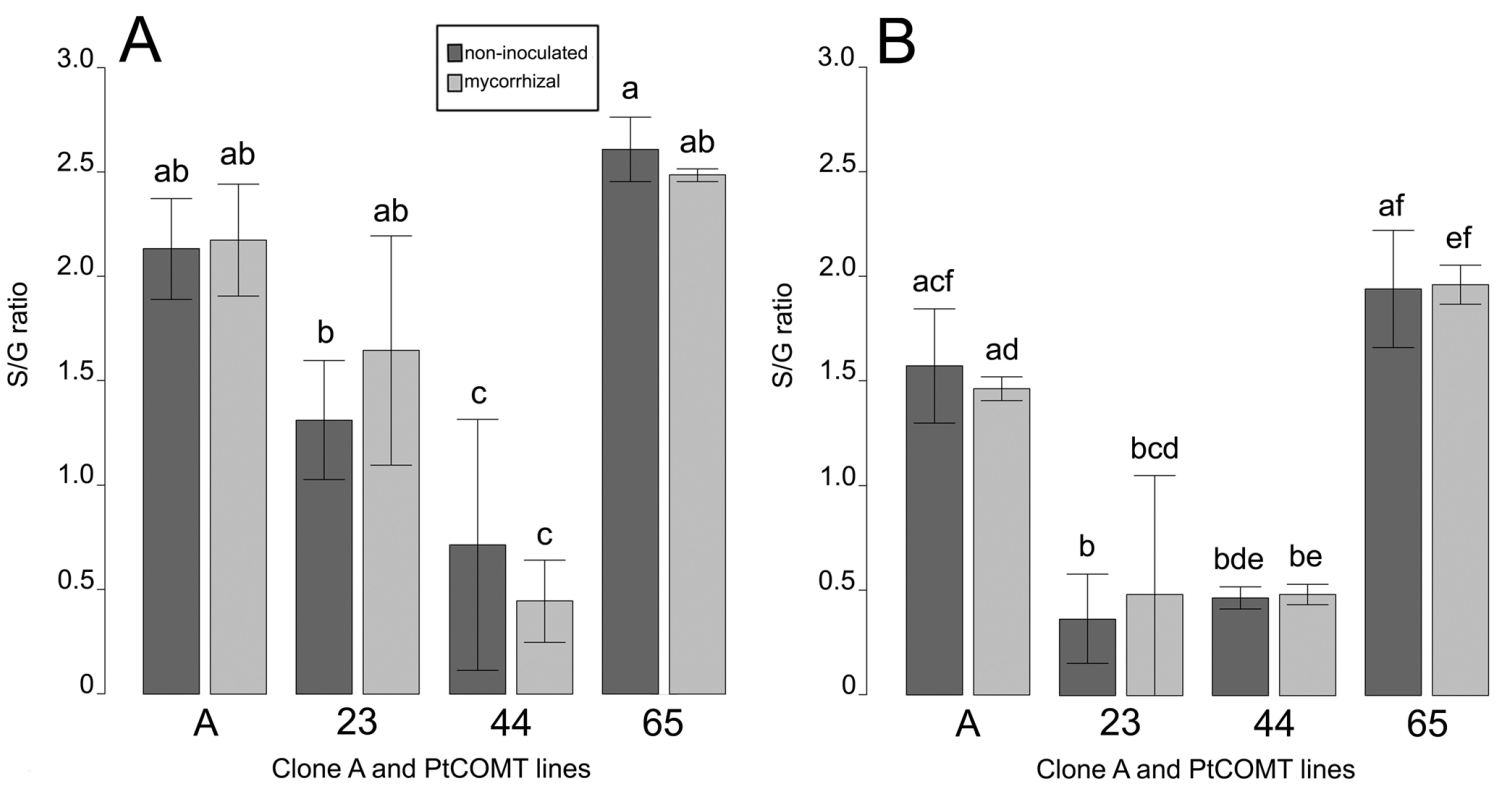
**The lignin syringyl/quiaicyl ratios of non-inoculated and mycorrhizal silver birches**. The lignin syringyl/quiaicyl (S/G) ratios of stems (A) and roots (B) of non-transgenic clone A and PtCOMT-modified lines 23, 44 and 65. Values are means ± standard deviation. Different letters above the columns denote significant (*P *< 0.05) differences between the non-inoculated and mycorrhizal plants within the line/clone and between lines/clone within the fungal treatment according to the Wilcoxon rank sum test with the Benjamini & Hochberg correction or the two-sample t-test with the Benjamini & Hochberg correction. Number of replicates 3.

**Figure 3:**
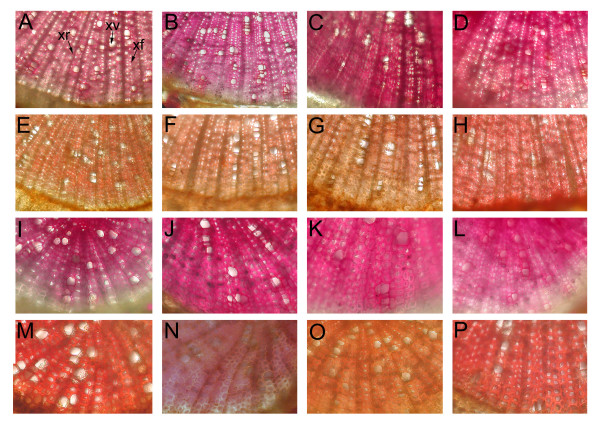
**Histochemical localization of lignin in non-inoculated silver birches**. Cross-sections of stem and root of non-inoculated clone A (A, E, I, M) and PtCOMT-modified lines 23 (B, F, J, N), 44 (C, G, K, O) and 65 (D, H, L, P). Stems (A-H) and roots (I-P). Lignin stained pink-red in the phloroglucinol-HCL stained sections (A-D, I-L). In the Mäule stainings (E-H, M-P), syringyl lignin pink-red and guaiacyl lignin light brown to dark brown. xf, xylem fibre; xr, xylem ray; xv, xylem vessel.

Lignin content as a percentage of dry weight (DW) evaluated with the acetyl bromide method was 27.6% in the non-inoculated and 27.1% in the mycorrhizal roots of clone A. In PtCOMT lines the root lignin content varied between the highest value of 27.8% of mycorrhizal roots of line 65 and the lowest of 24.5% of mycorrhizal roots of line 23. The corresponding lignin contents of stem wood were more than 5 percentage units lower than the root lignin contents and varied between 19.5 and 23.5%. Neither the transgene nor the fungal treatment affected the lignin content. The GC-MS analyses of lignin units showed that the non-inoculated clone A had higher (*P *< 0.05) S/G ratios in both stem and root wood than the non-inoculated plants of PtCOMT line 44 (Figure 2A and 2B). In non-inoculated roots of PtCOMT line 23 the S/G ratio was lower (*P *< 0.05) than in the roots of clone A. The S/G ratio of stem and root wood of mycorrhizal PtCOMT line 44 was significantly reduced (*P *< 0.05) in comparison with the mycorrhizal clone A. In the stem and root wood of both non-inoculated and mycorrhizal PtCOMT line 65, the S/G ratios were at the same level as in clone A. Moreover, in the stem and root wood of non-inoculated PtCOMT line 65, the S/G ratios were significantly (*P *< 0.05) higher than in the corresponding non-inoculated PtCOMT lines 23 and 44. According to the Mäule assay the S lignin (i.e. the pink-red colouration) was only slightly reduced when the root and stem xylem sections of PtCOMT lines 23 (Figure 3F, N) and 44 (Figure 3G, O) were compared to the xylem sections of clone A (3E, M) and PtCOMT line 65 (3H, P).

### Soluble phenolic compounds and condensed tannins

No clone- or line-specific peaks were detected in the HPLC-DAD or HPLC-MS chromatograms and, moreover, all phenolic compounds were present in the non-inoculated and mycorrhizal samples of clone A and PtCOMT lines (Table 1 and Additional file 3). Acetylated kaempherol, myricetin and quercetin with rhamnoside moiety were found in all leaves of clone A and PtCOMT lines. Condensed tannin concentrations were high in the samples (Table 1). The tannin levels partly prevented the identification of soluble phenolic components, especially from the root samples (Additional file 3).

**Table 1:** Concentrations of phenolic compounds and condensed tannins in non-inoculated and mycorrhizal silver birches

***Leaves***		**Clone**	**Lines**		
	**T**	**A**	**23**	**44**	**65**
Cinnamic acid derivatives	c	2.48 ± 0.44 a	2.12 ± 0.57 a	2.21 ± 0.37 a	2.30 ± 0.43 a
	ECM	2.84 ± 0.71 a	2.35 ± 0.72 a	2.35 ± 0.41 a	2.50 ± 0.54 a
Flavonoids	c	26.14 ± 4.00 a	30.67 ± 8.64 a	28.85 ± 2.14 a	30.87 ± 8.68 a
	ECM	27.07 ± 7.36 a	25.38 ± 9.78 a	25.80 ± 5.25 a	29.71 ± 5.80 a
Apigenin derivatives	c	0.20 ± 0.12 a	0.31 ± 0.10 a	0.31 ± 0.09 a	0.43 ± 0.25 a
	ECM	0.40 ± 0.21 a	0.19 ± 0.11 a	0.24 ± 0.07 a	0.39 ± 0.12 a
Kaempherol derivatives	c	0.85 ± 0.13 a	0.71 ± 0.23 a	0.90 ± 0.19 a	0.68 ± 0.20 a
	ECM	0.83 ± 0.14 a	0.73 ± 0.22 a	0.97 ± 0.13 a	0.69 ± 0.06 a
Myricetin derivatives	c	18.31 ± 3.58 a	22.81 ± 6.34 a	21.77 ± 1.75 a	21.66 ± 6.11 a
	ECM	18.83 ± 5.04 a	18.40 ± 8.20 a	17.90 ± 4.30 a	20.17 ± 4.54 a
Quercetin derivatives	c	5.19 ± 1.34 a	6.34 ± 2.12 a	5.43 ± 1.45 a	7.05 ± 1.64 a
	ECM	6.38 ± 1.52 a	5.52 ± 1.98 a	5.84 ± 1.18 a	7.20 ± 1.19 a
Condensed tannins	c	160.41 ± 24.97 a	142.87 ± 47.63 a	127.54 ± 34.23 a	154.82 ± 28.44 a
	ECM	142.40 ± 68.98 a	106.7 ± 22.57 a	149.21 ± 24.76 a	142.32 ± 62.23 a
*p*-OH-cinnamic acid derivatives	c	3.21 ± 0.47 a	1.84 ± 0.49 b	2.46 ± 0,32 ab	2.71 ± 0.61 ab
	ECM	3.10 ± 0.36 ac	2.22 ± 0.71 bc	2.42 ± 0.32 ac	2.42 ± 0.38 ab

***Stems***		**Clone**	**Lines**		
	**T**	**A**	**23**	**44**	**65**

Cinnamic acid derivatives	c	2.68 ± 0.68 a	1.37 ± 0.28 b	1.33 ± 0.25 b	1.65 ± 0.37 bc
	ECM	2.33 ± 0.33 ac	1.46 ± 0.18 b	1.27 ± 0.30 b	1.18 ± 0.20 b
Flavonoids	c	14.33 ± 1.74 ab	11.43 ± 1.49 ab	10.23 ± 1.43 b	13.79 ± 2.81 ab
	ECM	14.60 ± 2.12 a	12.38 ± 1.89 ab	10.77 ± 2.37 ab	11.18 ± 1.47 ab
Phenolic glycosides	c	16.86 ± 1.46 ab	19.48 ± 0.81 ab	18.18 ± 2.22 ab	22.67 ± 5.31 b
	ECM	15.40 ± 2.74 a	20.61 ± 2.29 ab	20.61 ± 2.29 ab	17.37 ± 5.29 ab
Condensed tannins	c	108.57 ± 50.76 a	138.57 ± 13.52 a	137.43 ± 13.72 a	141.47 ± 16.70 a
	ECM	144.82 ± 19.68 a	132.86 ± 11.23 a	131.90 ± 9.26 a	130.32 ± 12.55 a

***Roots***		**Clone**	**Lines**		
	**T**	**A**	**23**	**44**	**65**

Cinnamic acid derivatives	c	0.71 ± 0.33 a	0.17 ± 00.05 a	0.17 ± 0.10 a	0.37 ± 0.23 a
	ECM	0.53 ± 0.30 a	0.19 ± 0.02 a	0.20 ± 0.13 a	0.24 ± 0.14 a
Flavonoids	c	11.39 ± 0.62 a	9.45 ± 1.83 a	8.41 ± 1.93 a	8.26 ± 2.26 a
	ECM	11.24 ± 2.78 a	12.10 ± 2.55 a	7.88 ± 2.91 a	7.21 ± 1.25 a
Gallo/Ellagitannins	c	0.06 ± 0.01 a	0.32 ± 0.24 a	0.28 ± 0.09 a	0.12 ± 0.06 a
	ECM	0.11 ± 0.06 a	0.36 ± 0.08 a	0.23 ± 0.12 a	0.15 ± 0.13 a
Condensed tannins	c	130.50 ± 22.43 a	113.87 ± 17.86 a	106.92 ± 27.75 a	112.24 ± 8.94 a
	ECM	126.09 ± 6.80 a	116.24 ± 10.11 a	108.47 ± 5.62 a	100.46 ± 2.24 a
Condensed tannin precursors	c	35.07 ± 5.81 ab	31.44 ± 7.15 ab	28.60 ± 5.50 ab	28.50 ± 3.81 ab
	ECM	37.20 ± 9.43 ac	41.92 ± 10.62 a	20.38 ± 6.70 b	25.44 ± 2.21 bc

Concentrations (mg/DW g) of phenolic compounds and condensed tannins in the leaf, stem and root samples of silver birch clone A and PtCOMT-modified lines 23, 44 and 65 after 8 weeks in co-culture with *P. involutus*. Values are means ± standard deviations in the presence (ECM) or absence (c) of the fungus. Different letters following the values denote significant differences (*P *< 0.05) between the non-inoculated and mycorrhizal plants within the line/clone and between lines/clone within the fungal treatment according to the Kruskal-Wallis test combined with the Wilcoxon rank sum test with the Benjamini & Hochberg correction or the one-way or two-way Anova combined with Tukey's honestly significant difference test or with the two-sample t-test with the Benjamini & Hochberg correction. For statistical testing the leaf apigenin derivatives were square root, stem cinnamic acid derivatives log 10 and root condensed tannins square transformed. Number of replicates 4-7.

In the leaves of mycorrhizal plants, significant differences (*P *< 0.05) were found in the concentrations of quercetin 3-arabinose and kaempherol 3-acetyl-glucoside between PtCOMT lines 44 and 65 (Additional file 3). Significant differences between clone A and the PtCOMT lines were found in the concentration of *p*-OH-cinnamic acid derivates, individual cinnamic acid derivatives 3 and 4 and chlorogenic acid and chlorogenic acid derivative. A significant difference (*P *< 0.05) was detected in the amount of (+)-catechin in the leaves of the mycorrhizal and non-inoculated plants of clone A.

In stems, the cinnamic acid derivatives were at a higher (*P *< 0.05) level in clone A than in PtCOMT lines 23 and 44 (Table 1). Of individual components, the concentration of *p*-OH-cinnamic acid glucoside was higher (*P *< 0.05) in the stems of the clone A than in the stems of PtCOMT lines 23 and 44 (Additional file 3). Significant differences were also detected between the PtCOMT lines and clone A in the concentrations of gallocatechin, 3,4'-dihydroxypropiophenone 3-glucoside (DHPPG) and (+)-catechin, which was at a higher level in the stems of clone A than in the stems of PtCOMT lines.

The concentration of condensed tannin precursors was significantly (*P *< 0.05) higher in the mycorrhizal roots of line 23 than in the roots of lines 44 and 65 (Table 1). A small amount of ellagic acid was found in PtCOMT lines 23 and 44, where the PtCOMT was driven by the 35S promoter, but not in lines 65 and clone A (Additional file 3). An ellagic acid derivative was also found in line 65 and in the mycorrhizal roots of clone A.

### Formation of ECMs and growth characteristics of silver birches

**Figure 4:**
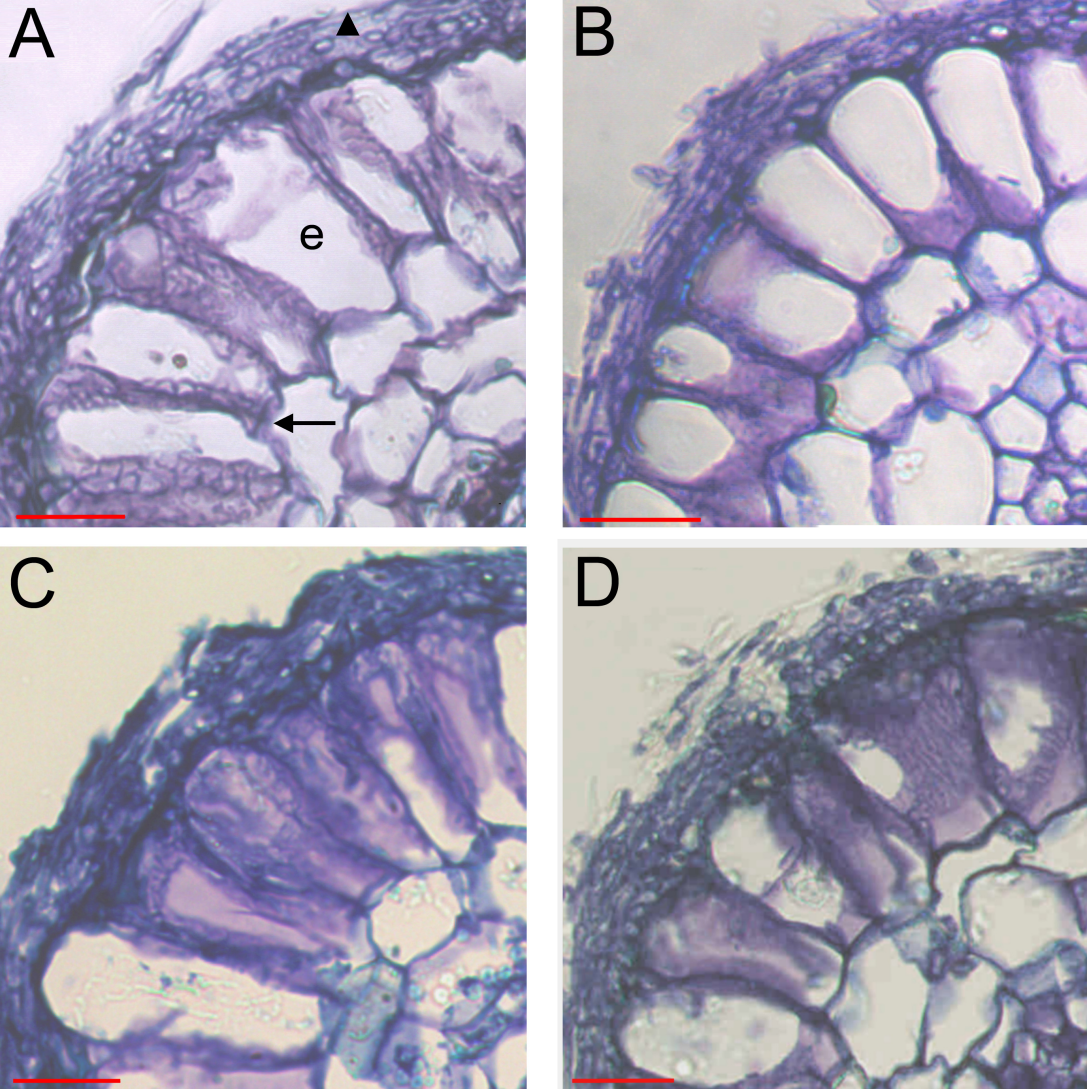
**ECM roots of silver birch**. Cross-sections (5-10 μm) of silver birch clone A (A) and PtCOMT lines 23 (B), 44 (C) and 65 (D) roots after 8 weeks of co-cultivation with *P. involutus*. Arrow, Hartig net; triangle, mycelium of *P. involutus*; e, epidermal cell of silver birch root. Bars = 20 μm.

All PtCOMT lines were able to form ECM symbiosis with *P. involutus*, and inoculation resulted in slightly higher survival percentages in clone A and PtCOMT lines 23 and 44 (Table 2). The mycorrhizal percentages of inoculated plants varied considerably between PtCOMT lines and clone A. No differences were detected in the number of ECMs per root systems (Table 2) or in the morphology of the mycorrhizas between lines: well-developed hyphal mantle covered the root tips and the epidermal cells were radially elongated and surrounded by fungal Hartig net (Figure 4A-D). Compared with the fresh weights (FWs) of the PtCOMT-modified lines, plants of clone A had lower FWs, but the growth rate (i.e. final FW/initial FW ratio) of the clone A plants in both treatments corresponded to the growth of the transgenic lines (Table 3). Inoculation of PtCOMT-modified lines with *P. involutus *enhanced their growth, resulting in significantly (*P *< 0.05) higher FWs than that of the non-inoculated plants (Table 3). The root/shoot ratios of plants increased significantly (*P *< 0.05) as a result of inoculation in clone A and PtCOMT line 23. Inoculation had no effect on the number or length of adventitious roots.

**Table 2:** Survival and ECM characteristics of silver birches

				**ECM categories**				
**Clone/** **Line**	**Survival % of non-inoculated plants**	**Survival % of inoculated plants**	**ECM %**	**I**	**II**	**III**	**IV**	**V**
A	74	76	31	3	2	1	2	1
23	92	95	83	11	8	3	4	3
44	95	100	74	14	2	6	3	3
65	92	87	58	8	5	3	1	2

Survival percentages of non-inoculated and inoculated silver birches of clone A and PtCOMT-modified lines 23, 44 and 65, percentages of the ECM plants of all inoculated plants (ECM %) and number of ECM root tips in root systems classified to five categories: I = 1-20 ECMs, II = 20-30 ECMs, III = 30-50 ECMs, IV = 50-100 ECMs, V ≥ 100 ECMs.

**Table 3:** Growth characteristics of non-inoculated and mycorrhizal silver birches

**Clone/** **Line**	**T**	**Initial FW (g)**	**Final FW (g)**	**Ratio of final and initial FW**	**Root/shoot FW ratio**	**Number of adventitous roots**	**Length of adventitous roots (cm)**
A	c	0.08 ± 0.04 a	1.75 ± 0.76 a	25.79 ± 19.90 a	0.80 ± 0.25 a	4.00 ± 1.33 a	16.70 ± 2.83 a
	ECM	0.07 ± 0.02 a	2.03 ± 0.76 a	34.49 ± 17.44 a	0.97 ± 0.24 b	4.00 ± 1.31 a	17.14 ± 1.04 a
							
23	c	0.15 ± 0.08 a	2.76 ± 0.88 a	23.75 ± 16.23 a	1.39 ± 0.40 a	4.97 ± 1.49 a	17.55 ± 3.78 a
	ECM	0.16 ± 0.09 a	3.06 ± 0.86 b	23.06 ± 13.63 a	1.59 ± 0.48 b	5.50 ± 1.72 a	17.59 ± 3.35 a
							
44	c	0.14 ± 0.06 a	2.70 ± 0.37 a	22.93 ± 9.28 a	1.40 ± 0.26 a	5.15 ± 1.54 a	18.64 ± 2.00 a
	ECM	0.15 ± 0.08 a	2.89 ± 0.51 b	23.28 ± 12.30 a	1.43 ± 0.29 a	7.68 ± 3.16 a	19.07 ± 2.10 a
							
65	c	0.17 ± 0.08 a	2.59 ± 0.43 a	17.70 ± 7.03 a	1.19 ± 0.20 a	4.03 ± 0.85 a	19.52 ± 2.05 a
	ECM	0.18 ± 0.09 a	2.83 ± 0.47 b	20.73 ± 11.90 b	1.26 ± 0.31 a	4.53 ± 1.22 a	18.52 ± 1.85 a

Effects of the mycorrhiza formation on the growth of silver birch control clone A and PtCOMT-modified lines 23, 44 and 65 after 8 weeks in co-culture with *P. involutu*s in a greenhouse. Initial fresh weights (FWs) and final FWs of plants, root/shoot ratios, number and length of adventitious roots. Values are means ± standard deviations in the presence (ECM) or absence (c) of the fungus. Different letters following the values denote a significant difference (*P *< 0.05) between the non-inoculated and inoculated plants with mycorrhizas within each line/clone according to the Wilcoxon rank sum test or the two-sample t-test. Number of replicates 9-35.

## Discussion

In the present study, no changes were found in phenolic compounds of PtCOMT-modified silver birch lines that would have been caused by the formation of ECM symbiosis with *P. involutus*. The only difference between the mycorrhizal and non-inoculated plants was observed in the catechin concentration in the leaves of the non-transgenic clone A. Münzenberger et al. [39,40] observed a reduction in various phenolic compounds (e.g. *p-*hydroxybenzoic acid glucoside, picein and catechin) in the mycorrhizal fine roots of European larch (*Larix decidua *Mill.) and Norway spruce [*Picea abies *(L.) Karst.] when compared with the non-mycorrhizal roots. Similar results were obtained with European beech (*Fagus sylvatica *L.) ECM roots which contained less catechin [41]. By contrast, an increase in catechin concentration was detected in the ECM roots of European larch [42] and the needles and stems of mycorrhizal Scots pine (*Pinus sylvestris *L.) [43]. Furthermore, enhanced levels of phenolic compounds have been observed in Douglas-fir [*Pseudotsuga menziesii *(Mirb.) Franco] [60] and brown barrel (*Eucalyptus fastigata *Deane and Maiden) [37]. The discrepancy of results may reflect the fluctuation of transcriptome patterns during ECM formation, as seen in various microarray experiments [32-34], diverse biological material and experimental designs. In the present work, all PtCOMT lines were able to form symbiosis with morphologically normal ECMs. Moreover, the mycorrhizal interaction increased FWs in all PtCOMT lines. Similar results have been obtained with silver birches expressing sugar beet chitinase IV [61] and 4-coumarate: coenzyme A ligase (*4CL*) [52] and *PtCOMT *[53]. In all of these studies, transgenic silver birches were capable of forming ECM symbiosis although *4CL *expressing silver birches had changes in their growth characteristics [52] and two PtCOMT silver birch lines had altered ECM morphology *in vitro *[53].

In silver birch-*P. involutus *interaction, Feugey et al. [62] observed a transient increase in phenylalanine ammonia-lyase (PAL) activity, but in the micro-array studies [31,33] *PAL *was not differentially expressed in ECM roots compared with non-inoculated roots. Instead, Le Quéré et al. [33] found an increase in genes coding monolignol biosynthesis route associated products: *Arabidopsis *caffeoyl-coA 3-*O*-methyltransferase (CCoAOMT) homolog, dirigent protein homolog and sinapyl alcohol dehydrogenase (SAD) homolog. *CCoAOMT *expression was consistent after 4 days of inoculation to 14 days, whereas the expression of dirigent protein homolog and *SAD *homolog was at its highest after 2 days of inoculation and then again 14 days after the start of the co-cultivation. Our results indicate that ECM formation had no drastic effect on the lignin or the phenolic compound biosynthesis in stems or roots.

The expression of *PtCOMT *under control of the 35S promoter resulted in lower S/G ratios in the stem and root wood when compared with clone A, as observed in previous studies [48,53,63]. By contrast, when *PtCOMT *was under the UbB1 promoter, no changes were detected in the lignin characteristics. We have previously shown [48,63] that there are multiple copies of the *PtCOMT *gene in lines 23, 44 and 65 and that the UbB1-*PtCOMT*-transcript is bigger than the 35S-*PtCOMT*-transcript. In the present study, the relative expression of the heterologous *PtCOMT *seemed to be higher in the roots of 35S-PtCOMT lines 23 and 44 than in those of UbB1-PtCOMT line 65. Conversely, the *BpCOMT *mRNA transcript levels were more decreased in 35S-PtCOMT lines 23 and 44 than in UbB1-PtCOMT-line 65. The homology between *BpCOMT *and *PtCOMT *at the nucleotide level was quite high and it is therefore possible that the heterologous *PtCOMT *expression resulted in RNAi-mediated partial silencing of the endogenous *BpCOMT*. The relative expression levels of *BpCOMT *and *PtCOMT *possibly indicate that the 35S promoter generated a higher number of mRNA transcripts of *PtCOMT *than UbB1 and, as a consequence, decreased the number of *BpCOMT *transcripts more intensively in 35S-PtCOMT lines 23 and 44, thus causing a reduction in the lignin S/G ratio.

The monolignol biosynthetic pathway crosstalks with other cell wall associated pathways [64,65] and also with the biosynthetic pathways of various phenolic compounds [66-70] which share the same precursors. Consequently, the altered expression of monolignol biosynthetic pathway genes may result in changes in the lignin content and phenolic compound profiles as shown with suppressed COMT and CCoAOMT (EC 2.1.1.104) [66], cinnamoyl-CoA reductase (CCR; EC 1.2.1.44) [68], hydroxycinnamoyl-CoA shikimate/quinate hydroxycinnamoyl transferase (HCT; EC 2.3.1.133) [69] and cinnamate 4-hydroxylase (C4H; 1.14.13.11) [67,70]. In the present study, chemical changes were detected between the PtCOMT lines and the non-transgenic clone A in the concentrations of phenolic compounds in the roots, stems and leaves of both non-inoculated and mycorrhizal plants. The detected changes were probably not direct results of the transgene because 35S-PtCOMT lines 23 and 44 displayed differences in phenolic compound profiles. Furthermore, the changes in the phenolic profiles of leaves, stems and roots are within the natural variation of phenolic compounds within silver birch [1-3,5,7].

## Conclusion

In the present study, the down-regulation of *BpCOMT *in the 35S-PtCOMT lines caused no shift of monolignol pathway intermediates to the biosynthesis of the phenolic secondary compounds. Moreover, no apparent effect in the composition or quantity of phenolic compounds caused by the expression of *PtCOMT *under the 35S or UbB1 promoter could be found. To conclude, our results indicate that the present lignin modification in the PtCOMT lines does not affect phenolic profiles or the symbiotic relationship between silver birch and *P. involutus*.

## Methods

### Plant and fungal material

Silver birch (*Betula pendula *Roth.) lines 23, 44 and 65 expressing the caffeate/5-hydroxyferulate *O*-methyltransferase (*PtCOMT*) gene [EMBL: X62096] of quaking aspen (*Populus tremuloides *L.) [17] were generated as described by Aronen et al. [63] and Tiimonen et al. [48]. The *PtCOMT *encodes Class II methyltransferase (EC 2.1.1.68), which uses 5-hydroxyconiferyl aldehyde as a primary substrate [14]. All transgenic lines were produced from clone A as described in Valjakka et al. [71], originating in Punkaharju, Eastern Finland (61°48' N, 29°17' E). In PtCOMT lines 23 and 44, the transgene was driven by the 35S cauliflower mosaic virus (CaMV) promoter and in line 65 by the sunflower polyubiquitin (UbB1) promoter. The gene constructs were pRT99/35S-*PtCOMT *and pRT99/UbB1-*PtCOMT*, respectively. The plants of all lines were multiplied on Woody Plant Medium (WPM) [72] containing 2.2 μM 6-benzyladenine (BA) and 2.8 μM indole-3-acetic acid (IAA) and subsequently rooted for 6 weeks on the same media without any growth regulators. The rooted plants were acclimated for 2 weeks in a sterile peat-vermiculite (1:10; v:v) mixture moistened with modified Melin-Norkrans nutrient solution (MMN) [73] (3.7 mM KH_2_PO_4_, 1.9 mM (NH_4_)_2_HPO_4_, 0.45 mM CaCl_2_, 0.43 mM NaCl, 0.61 mM MgSO_4 _• 7H_2_O, 0.2 μM thiamine-HCl, 18.4 μM FeCl_3 _• 6H_2_O, pH 5.8) without glucose.

The ECM fungus *Paxillus involutus *(Batsch: Fr.) strain (ATCC 200175) was maintained by cultivating the mycelium on Hagem agar medium [74] in darkness at 21°C. For the experiment, the mycelium was cultivated for 2 weeks on the same medium.

### Co-cultivation of silver birches and *P. involutus*

Before starting the co-cultivation, individual silver birches were weighed and photographed and the number of adventitious roots was monitored. For the co-cultivation, the root system of a plant was transferred to a Petri dish, 14 cm in diameter and filled with a sterile peat-vermiculite (1:10; v/v) mixture moistened with modified MN nutrient solution without glucose. The shoot was positioned outside the Petri dish through an opening in the sidewall of the dish. Three mycelial agar plugs cut from a 2-week-old culture of *P. involutus *were placed close to the roots of individual plants. Plain agar plugs were used as a substitute for mycelial agar plugs in the non-inoculated treatments. The number of replicates per fungal treatment and line was 38. The Petri dishes were closed with parafilm and brown paper was attached to each dish lid. The co-cultivation took place in a greenhouse at the Botanical Garden of the University of Oulu under a 16-h photoperiod (340-580 μE m^-2 ^s^-1^, high pressure sodium lamps, Master SON-T PIA Plus 400 W, Philips, Amsterdam, Netherlands) at 20°C in a randomly assigned design. Relative humidity in the greenhouse was 88. From the second co-cultivation week on, plants were treated twice a week with 3% pine soap. Five weeks after inoculation, water and 10 ml of modified half-strength MN nutrients were added to all cultivations. The plants were cultivated with the fungus for 8 weeks. At harvest, the shoots and roots were weighed and the number of adventitious and lateral roots was measured. All root systems were evaluated with a dissecting microscope and the ECM status of the mycorrhizal plants was categorized into five classes (I-V) according to the number of ECM root tips per a root system (I; 1-20, II; 20-30, III; 30-50, IV; 50-100, V; more than 100 ECMs per root system).

### Sequencing of silver birch *COMT *and *PP2A*

Wood of clone A was ground in liquid nitrogen and RNA extracted using the method described by Jaakola et al. [75] and quantified with a ND-1000 UV-Vis spectrophotometer (NanoDrop Technologies, Wilmington, USA). SuperScript II reverse transcriptase (Invitrogen, Carlsbad, CA, USA) was used to prepare cDNA from 2 μg of total RNA in a standard reaction with anchored oligo-dT primers. The PCR amplification of fragments was performed using DyNAzyme™II polymerase (Finnzymes, Finland) and degenerated primers 5'-ATGGG(GATC)TC(GCA)AC(AC)(AG)(GC)(GATC)GA(AG)AC-3' as a forward and 5'-(AG)(GATC)GT(AG)TTG(AT)A(GATC)GCA(GC)A(AG)CAC-3' as a reverse for putative silver birch *COMT *and primers 5'-GATGATGATGAGGTACTTCTTGCG-3' as a forward and 5'-ATTTGATGTTTGGAACTCTGTC-3' as a reverse for protein phosphatase 2A regulatory subunit (*PP2A*). The 3' ends of *COMT *and *PP2A *were amplified with the SMART™ RACE cDNA amplification Kit (Clontech Laboratories, Palo Alto, CA) following the instructions of the manufacturer. The gene-specific primers for 3'-RACE PCR reactions were 5'-CGCGGAAACTCAGATGACTCCAACTCAA-3' and 5'-GCTTGCGGAGGATAGGCATTGGAGAGTA-3' for *COMT *and *PP2A*, respectively. The PCR products were gel purified with NucleoSpin Extract (Macherey-Nagel, Düren, Germany). Fragments of putative putative *PP2A *were sequenced directly from the PCR product and the *COMT *were subcloned using a Qiagen PCR Cloning Kit (Germantown, MD, USA). The sequences were determined with an ABI PRISM 377 DNA sequencer (Perkin-Elmer, Wellesley, MA, USA) and a BigDye Terminator v3.1 Cycle Sequencing Kit (Applied Biosystems, Foster City, CA, USA). Three to five plasmids were sequenced per putative *COMT *fragment.

### Relative quantification of *PtCOMT *and silver birch *COMT *mRNAs

The root samples from the non-inoculated and mycorrhizal plants representing all transgenic lines and control clone A were ground in liquid nitrogen. RNA from the samples was extracted with an E.Z.N.A.^® ^Plant RNA Kit (Omega Bio-Tek Inc., Doraville, GA, USA) following the manufacturer's instructions, and its quality and quantity were checked with an agarose gel electroforesis and a ND-1000 UV-Vis spectrophotometer (NanoDrop Technologies). cDNA was prepared with SuperScript II reverse transcriptase (Invitrogen) using 300 ng of total RNA. All cDNAs were gel-purified using a DNA Gel Extraction Kit (Millipore Corporation, Billerica, MA, USA), and cDNA acquisitions were determinated with a ND-1000 UV-Vis spectrophotometer. The real-time PCR reactions consisted of LightCycler 480 SYBR green 1 Master mix (Roche, Meylan, France) and 0.50 μM each primer and were run with a LightCycler ^® ^480 system (Roche, Penzberg, Germany). The primers used were: *atub *5'-AATGCGTGCTGGGAACT-3' (forward) and 5'-GATGACAGTGGGTTCCAGAT-3' (reverse); *BpCOMT *5'-CCAGATGCACCAGTTATGCT-3' (forward) and 5'-GAGCAGCAATAGACACACCA-3' (reverse); *BpPP2A *5'-GGAGGATAGGCATTGGAGAG-3' (forward) and 5'-CTGCATCACGGATCGAGTAA-3' (reverse); *PtCOMT *5'-GCCATTGAACTCGACCTT-3' (forward) and 5'-AGATCTTTCAGAGAGCAGGTAA-3' (reverse). The real-time PCR amplification cycles were as follows: incubation at 95°C for 10 min followed by 35 cycles: 10 s at 95°C, 10 s at 60°C and 5 s at 72°C. Each sample was run as a duplicate, and the number of biological replicates was 4 or 5 per line and fungal treatment. The PCR products were analysed using the melting curve analysis of LightCycler 480 software release 1.5 (Roche) and the specificity of all the primers were confirmed by sequencing the product of RT-PCR. Products were first purified according the instructions of NucleoSpin Extract (Macherey-Nagel) and then directly sequenced using BigDye Terminator v3.1 chemistry (Applied Biosystems) with the ABI PRISM 377 DNA sequencer (Perkin-Elmer). The quantification of the target genes was conducted using a calibrator-normalized procedure with the alpha-tubulin (*atub*) gene [GenBank: AJ279695] of silver birch and the putative *PP2A *[76,77] of silver birch as reference genes. A primer pair specific (*BpCOMT, PtCOMT, BpPP2A *and *atub*) standard curve of amplification efficiency was used in the calculation of the relative amount of target (*BpCOMT *and *PtCOMT*) and reference genes (*atub *and the putative *BpPP2A *of silver birch), generated with dilutions of pooled silver birch cDNA samples. The calibrator normalized relative expression was determined as a ratio between the relative amount of target (*BpCOMT *and *PtCOMT*) and reference (*BpPP2A *and *atub*) genes normalized e.g. divided by the target/reference ratio of the calibrator (Roche Applied Science Technical Note No. LC 13/2001).

### Quantification of lignin content

The root and stem samples from the non-inoculated and inoculated plants representing all transgenic lines and control clone A were first dried at 60°C for 72 h and then the bark was removed and wood was ground to fine powder. Powdered samples of 5 mg were extracted with acetone, and lignin contents were determined with an acetyl bromide method as described by Koutaniemi et al. [78] from three biological replicates. Klason lignin of the pooled root and stem samples was used as a standard in the equation that was employed in the calculation of lignin contents. Klason lignin was determined gravimetrically from the barked and homogenized samples as described by Tiimonen et al. [48]. Two parallel determinations were carried out per pooled sample. The Klason lignin contents in the root and stem samples were 26.06 and 21.44% of DW, respectively.

### Determination of syringyl and guaiacyl moieties

Syringyl (S) and guaiacyl (G) monomers of the root and stem lignin were analyzed from dried (60°C for 72 h), barked and ground samples, using the modified method of thioacidolysis [79]. A sample of 1-3 mg was extracted with ethanol before thioacidolysis, which was conducted from three biological replicates. The thioacidolysis procedure and the chromatographic conditions were conducted as described by Tiimonen et al. [48]. Based on the individual mass spectra, peak areas of two ions were used in the selective ion monitoring (SIM) analyses. Selected ions were m/z 269 for G-units and m/z 299 for S-units.

### Analyses of phenolic compounds and condensed tannins

The leaf, root and stem samples from the non-inoculated and inoculated plants representing all transgenic lines and control clone A were dried at 60°C for 72 h and stored at -20°C until analyses. Four to six replicates of the leaf, root and stem samples (8 mg, 15 mg and 20 mg, respectively) were homogenized with an Ultra-Turrax T8 homogenizer in 700 μl of methanol for 30 s. The samples were incubated in an ice bath for 15 min and centrifuged at 16 000 g for 3 min. Supernatants were collected and methanol extractions were repeated three more times with 5 min incubations on ice bath. The supernatants were combined and methanol evaporated under nitrogen. The extraction residues were dried at room temperature for 2 days for further analysis of tannins.

The samples were dissolved in 600 μl of water:methanol (1:1, v/v) and analyzed by HPLC (Agilent 1100 Series HPLC, Palo Alto, CA, USA) with a diode array detector (DAD). A hypersil ODS HPLC-column (4.6 mm × 60 mm, 3 μm particles, Hewlett-Packard, Germany) was used in the separation. The injection volumes of the leaf, root and stem samples were 20 μl, 15 μl and 10 μl, respectively. The compounds were identified and quantified based on their retention times, spectral characteristics and HPLC-MS (API-ES, positive ions) [80]. HPLC-MS (API-ES, pos. ions) produced the following molecular ions: kaempherol-acetylrhamnoside, (M+1) 474; myricetin-acetylrhamnoside, (M+1) 506; quercetin-acetylrhamnoside, (M+1) 490.

Quantification was conducted using following standards: apigenin (Roth, Karlsruhe, Germany) for the apigenin derivatives, (+)-catechin (Aldrich, Steinheim, Germany) for the catechin derivatives, chlorogenic acid (Aldrich) for the chlorogenic acid and cinnamic acid derivatives, gallic acid (Aldrich) for the gallotannins, kaempferol 3-*O*-glucoside (Extrasynthese, Genay, France) for the kaempferol derivatives, luteolin (Roth) for the luteolin derivatives, myricetin 3-rhamnoside (Apin Chemicals Ltd, Abingdon, UK) for the myricetin derivatives, picein (Extrasynthese) for 3,4'-dihydroxypropiophenone 3-glucoside (DHPPG), quercetin 3-galactoside (Roth) for the quercetin derivatives and salicin (Roth) for the condensed tannin precursors. The quantification of isorhamnetin 3-glucoside, platyfylloside, rhododendrin and salidroside was based on their own reference coefficients.

Soluble condensed tannins were determinated from the HPLC sample and insoluble condensed tannins from the dried extract residue by acid butanol assay [81]. The quantification of condensed tannins was based on purified tannin from dwarf birch (*Betula nana *L.).

### Toluidine blue staining of ECM roots

After analysis under a dissecting microscope, the mycorrhizal root tips were further examined by light microscopy. The root tips were fixed with 4% paraformaldehyde in 0.1 M phosphate-buffered saline (PBS) buffer (137 mM NaCl, 2.7 mM KCl, 8.0 mM Na2HPO4, 1.7 mM KH2PO4, pH 7.4) after which they were dehydrated in graded ethanol series, treated with 2-methyl-2-propanol and embedded into paraffin (Merck, Whitehouse Station, NJ, USA) blocks. Both longitudinal and cross-sections of 5-10 μm were used for staining with a 0.05% toluidine blue O solution. The root sections were examined with a light microscope (Nikon Optiphot 2, Japan) and imaged with an Infinity*1*-*3*C camera (Lumenera Corporatiom, Ottawa, Ontario, Canada), using the IMT iSolution Lite image-processing program (IMT i-Solution Inc., Vancouver, BC, Canada).

### Histochemical lignin staining

Hand-cut cross-sections were made of the upper parts of the roots and the bases of the stem. Phloroglucinol-HCL and Mäule staining assays were conducted as described in Guo et al. [82] with an additional potassium iodide treatment of sections at the end of the phloroglucinol-HCl staining. The samples were examined under a light microscope (Nikon Optiphot 2) and photographed with a digital camera (Nikon Coolpix 950, Japan).

### Statistical analysis

Statistical analyses were performed with an R software package 2.5.1 [83] and a graphical user interface, the R Commander [84].

Comparisons of growth characteristics between the non-inoculated and inoculated plants with mycorrhizas, i.e. mycorrhizal plants, within each line/clone were analysed with the Wilcoxon rank sum test or the two-sample t-test. Differences in the number of individual ECM root tips in the root systems between control clone A and PtCOMT lines 23, 44 and 65 were studied using Fisher's exact test.

The lignin quantity and S/G ratios were statistically tested using the Wilcoxon rank sum test or the two-sample t-test. The tests were performed between the non-inoculated and mycorrhizal plants within the line/clone and also between lines/clone within the fungal treatment. Phenolic compound and condensed tannin data was studied using a parametric one-way or two-way Anova combined with Tukey's honestly significant difference test or two-sample t-test or non-parametric Kruskal-Wallis test [85] combined with the Wilcoxon rank sum test when the assumptions of parametric tests were not met. Statistical testing was conducted between the non-inoculated and mycorrhizal plants within the line/clone or between lines/clone within the treatments. Square, square root, log 10 or inverse transformations were conducted to some of the variables of phenolic compounds. The Benjamini & Hochberg false discovery rate (FDR) [86] controlling the expected proportion of type I errors was used in the correction of multiple pairwise comparisons of lignin characteristics and phenolic data with the cut-off value of 0.05 [87,88].

The relative quantification of genes was performed using the ratio of reference gene amplification efficiency and amplification efficiency of the target gene. Averages obtained with both reference genes (*atub *and *PP2A*) were used in the statistical testing of gene expression with the Wilcoxon rank sum test with the Bonferroni correction or the two-sample t-test with the Bonferroni correction. The relative expression of *BpCOMT *was compared between the PtCOMT lines and clone A within the treatments. The relative expression of *PtCOMT *was statistically examined between the PtCOMT lines within the treatment.

## Accession Numbers

The open reading frame of silver birch (*Betula pendula *Roth) *COMT *(*BpCOMT*) and partial *PP2A *(*BpPP2A*) sequence can be found in the GenBank at the NCBI under accession numbers [GenBank: FJ667539] and [GenBank: FJ667540], respectively.

## Authors' contributions

KN and HH conceived the study. SS designed the study with KN and HH; carried out the experiment with KN, JE, JV, JK, RM and MS; conducted the molecular studies; did the stainings with JE; conducted the HPLC analysis with JE under the supervision of RJ-T and drafted the manuscript with KN, HT, VC, RJ-T and HH. TL optimized methods used in the lignin analysis and conducted analysis in co-ordination with PS. All authors read the manuscript and agree with the content.

## Supplementary Material

Additional file 1 **Alignment of predicted amino acid sequence of putative silver birch COMT**. Alignment of the putative silver birch caffeate/5-hydroxyferulate *O*-methyltransferase (*BpCOMT*) amino acid sequence with the COMT sequences of *Rosa chinensis *[EMBL: CAD29457], quaking aspen (*Populus tremuloides *L.) [EMBL: X62096], *Medicago sativa *[GenBank: M63853] and *Arabidopsis thaliana *[GenBank: NM_124796]. Conserved amino acids present in all sequences are highlighted in indigo blue and similar with blue-grey. Click here for file

Additional file 2 **Alignment of predicted amino acid sequence of putative silver birch PP2A**. Alignment of the partial silver birch protein phosphatase 2A regulatory subunit (*BpPP2A*) amino acid sequence with the PP2Asequences of *Medicago sativa *subsp. x *varia *[GenBank: [AAG29593]], *Arabidopsis thaliana *[GenBank: [NP_172790]], *Zea mays *[GenBank: [NP_001105839]] and *Oryza sativa *[EMBL: [CAB51803]]. Conserved amino acids present in all sequences are highlighted in indigo blue and similar with blue-grey. Click here for file

Additional file 3 **Concentrations of individual phenolic compounds**. Individual phenolic compounds (mg/DW g) identified from leaf, stem and root samples of non-inoculated and mycorrhizal silver birches of clone A and PtCOMT-modified lines 23, 44 and 65 after 8 weeks in co-culture with *P. involutus*. Values are concentration mg/DW g means ± standard deviations in the presence (ECM) or absence (c) of the fungus. Different letters following the values denote significant differences (*P *< 0.05) between non-inoculated and mycorrhizal plants within the line/clone and between lines/clone within the fungal treatment according to the Kruskal-Wallis test combined with the Wilcoxon rank sum test with the Benjamini & Hochberg correction or the one-way or two-way Anova combined with Tukey's honestly significant difference test or with the two-sample t-test with the Benjamini & Hochberg correction. Square root transformation was conducted to the chlorogenic acid, dicoumaroyl-astragalin and hyperin of leaves and isorhamnetin 3-glucoside of stems. Log 10 transformation was conducted to the chlorogenic acid derivatives of leaves and *p*-OH-cinnamic acid glucoside of stems. The inverse transformation was conducted to the cinnamic acid derivative 4 of leaves and salidroside of stems. Number of replicates 4-7. RT, retention time (min); nm, wavelength used in monitoring of the component. Click here for file
