# Supplementary material for: Phenolic compounds in ectomycorrhizal interaction of lignin modified silver birch
Source: BMC Plant Biol. 2009 Sep 29;9:124. doi: 10.1186/1471-2229-9-124 (PMC2763875; doi:10.1186/1471-2229-9-124)
Supplement: Additional file 1 — Alignment of predicted amino acid sequence of putative silver birch COMT. Alignment of the putative silver birch caffeate/5-hydroxyferulate O-methyltransferase (BpCOMT) amino acid sequence with the COMT sequences of Rosa chinensis [EMBL: CAD29457], quaking aspen (Populus tremuloides L.) [EMBL: X62096], Medicago sativa [GenBank: M63853] and Arabidopsis thaliana [GenBank: NM_124796]. Conserved amino acids present in all sequences are highlighted in indigo blue and similar with blue-grey. [file 1471-2229-9-124-S1.PDF]

```
                          *        20         *        40         *
B. pendula      MGSTAETQMTPTQVSDEEANLFAMQLASASVLPMILKSAIELDLLEIMAK
R. chinensis    MGSTGETQMTPTQVSDEEANLFAMQLASASVLPMVLKAAIELDLLEIMAK
P. tremuloides  MGSTGETQMTPTQVSDEEAHLFAMQLASASVLPMILKTAIELDLLEIMAK
M. sativa       MGSTGETQITPTHISDEEANLFAMQLASASVLPMILKSALELDLLEIIAK
A. thaliana     MGSTAETQLTPVQVTDDEAALFAMQLASASVLPMALKSALELDLLEIMAK

                         60         *        80         *       100
B. pendula      AGPGAYLSPSEIASQLPTTNPDAPVMLDRILRLLASYSVLTYSLRTLPDG
R. chinensis    AGPGAFLSPNDLASQLPTKNPEAPVMLDRMLRLLASYSILTYSLRTLPDG
P. tremuloides  AGPGAFLSTSEIASHLPTKNPDAPVMLDRILRLLASYSILTCSLKDLPDG
M. sativa       AGPGAQISPIEIASQLPTTNPDAPVMLDRMLRLLACYIILTCSVRTQQDG
A. thaliana     NGSPMSPTE--IASKLPTKNPEAPVMLDRILRLLTSYSVLTCSNRKLSGD

                          *       120         *       140         *
B. pendula      RVERLYGLGPVCKFLTKNEDGVSIAALNLMNQDKVLMESWYYLKDAVLEG
R. chinensis    KVERLYGLGPVCKFLTKNEDGVSIAALCLMNQDKVLVESWYHLKDAVLDG
P. tremuloides  KVERLYGLAPVCKFLTKNEDGVSVSPLCLMNQDKVLMESWYYLKDAILDG
M. sativa       KVQRLYGLATVAKYLVKNEDGVSISALNLMNQDKVLMESWYHLKDAVLDG
A. thaliana     GVERIYGLGPVCKYLTKNEDGVSIAALCLMNQDKVLMESWYHLKDAILDG

                        160         *       180         *       200
B. pendula      GIPFNKAHGMTSFEYHGKDLRFNKVFNKGMSDHSTITMKKILETYKGFEG
R. chinensis    GIPFNKAYGMTAFDYHGTDPRFNKVFNKGMADHSTITMKKILETYKGFEG
P. tremuloides  GIPFNKAYGMTAFEYHGTDPRFNKVFNKGMSDHSTITMKKILETYKGFEG
M. sativa       GIPFNKAYGMTAFEYHGTDPRFNKVFNKGMSDHSTITMKKILETYTGFEG
A. thaliana     GIPFNKAYGMSAFEYHGTDPRFNKVFNNGMSNHSTITMKKILETYKGFEG

                          *       220         *       240         *
B. pendula      LTSVVDVGGGTGAVLSMIVSKYPSIRGINFDLPHVIEDAPSYPGVDHVGG
R. chinensis    LTSIVDVGGGTGAVVNMIVSKYPSIKGINFDLPHVIEDAPQYPGVQHVGG
P. tremuloides  LTSLVDVGGGTGAVVNTIVSKYPSIKGINFDLPHVIEDAPSYPGVEHVGG
M. sativa       LKSLVDVGGGTGAVINTIVSKYPTIKGINFDLPHVIEDAPSYPGVEHVGG
A. thaliana     LTSLVDVGGGIGATLKMIVSKYPNLKGINFDLPHVIEDAPSHPGIEHVGG

                        260         *       280         *       300
B. pendula      DMFVSVPKGDAIFMKWICHDWSDEHCLKFLKNCYDALPNNGKVIVAECIL
R. chinensis    DMFVSVPKGDAIFMKWICHDWSDEHCLKFLKNCYAALPDNGKVILGECIL
P. tremuloides  DMFVSVPKADAVFMKWICHDWSDAHCLKFLKNCYDALPENGKVILVECIL
M. sativa       DMFVSIPKADAVFMKWICHDWSDEHCLKFLKNCYEALPDNGKVIVAECIL
A. thaliana     DMFVSVPKGDAIFMKWICHDWSDEHCVKFLKNCYESLPEDGKVILAECIL

                          *       320         *       340         *
B. pendula      PVAPDTSLATKGVIHIDVIMLAHNPGGKERTEKEFEALAKGAGFQGFQVL
R. chinensis    PVAPDTSLATKGVVHIDVVMLAHNPGGKERTEQEFEALAKGSGFQGIRVA
P. tremuloides  PVAPDTSLATKGVVHVDVIMLAHNPGGKERTEKEFEGLAKGAGFQGFEVM
M. sativa       PVAPDSSLATKGVVHIDVIMLAHNPGGKERTQKEFEDLAKGAGFQGFKVH
A. thaliana     PETPDSSLSTKQVVHVDCIMLAHNPGGKERTEKEFEALAKASGFKGIKVV

                        360
B. pendula      GCAFNTYIMEFIKKL--   :
R. chinensis    CNAFNTYVIEFLKKI--   :
P. tremuloides  CCAFNTHVIEFRKKA--   :
M. sativa       CNAFNTYIMEFLKKV--   :
A. thaliana     CDAFGVNLIELLKKL--   :
```
