# Supplementary material for: Phenolic compounds in ectomycorrhizal interaction of lignin modified silver birch
Source: BMC Plant Biol. 2009 Sep 29;9:124. doi: 10.1186/1471-2229-9-124 (PMC2763875; doi:10.1186/1471-2229-9-124)
Supplement: Additional file 3 — Concentrations of individual phenolic compounds. Individual phenolic compounds (mg/DW g) identified from leaf, stem and root samples of non-inoculated and mycorrhizal silver birches of clone A and PtCOMT-modified lines 23, 44 and 65 after 8 weeks in co-culture with P. involutus. Values are concentration mg/DW g means ± standard deviations in the presence (ECM) or absence (c) of the fungus. Different letters following the values denote significant differences (P < 0.05) between non-inoculated and mycorrhizal plants within the line/clone and between lines/clone within the fungal treatment according to the Kruskal-Wallis test combined with the Wilcoxon rank sum test with the Benjamini & Hochberg correction or the one-way or two-way Anova combined with Tukey's honestly significant difference test or with the two-sample t-test with the Benjamini & Hochberg correction. Square root transformation was conducted to the chlorogenic acid, dicoumaroyl-astragalin and hyperin of leaves and isorhamnetin 3-glucoside of stems. Log 10 transformation was conducted to the chlorogenic acid derivatives of leaves and p-OH-cinnamic acid glucoside of stems. The inverse transformation was conducted to the cinnamic acid derivative 4 of leaves and salidroside of stems. Number of replicates 4-7. RT, retention time (min); nm, wavelength used in monitoring of the component. [file 1471-2229-9-124-S3.PDF]

Leaves				Clone				Lines											
	RT	nm	T	A				23				44				65			
Apigenin derivative	39.8	320	c	0.05	±	0.06	a	0.07	±	0.06	a	0.04	±	0.04	a	0.04	±	0.03	a
			ECM	0.12	±	0.10	a	0.04	±	0.03	a	0.06	±	0.06	a	0.05	±	0.07	a
Apigenin glycoside	12.3	320	c	0.12	±	0.02	a	0.14	±	0.05	a	0.18	±	0.03	a	0.17	±	0.05	a
			ECM	0.13	±	0.03	a	0.11	±	0.05	a	0.12	±	0.02	a	0.19	±	0.06	a
(+)-Catechin	7.5	220	c	1.00	±	0.29	a	0.16	±	0.23	ab	0.18	±	0.25	ab	0.31	±	0.44	ab
			ECM	0.28	±	0.27	b	0.19	±	0.25	ab	0.16	±	0.20	ab	0.45	±	0.44	ab
Chlorogenic acid	7.9	320	c	0.56	±	0.25	ab	0.62	±	0.20	ab	0.61	±	0.17	ab	0.74	±	0.24	ab
			ECM	0.42	±	0.15	a	0.90	±	0.36	ab	0.78	±	0.31	ab	0.98	±	0.38	b
Chlorogenic acid derivative	12.0	320	c	0.14	±	0.02	a	0.08	±	0.02	b	0.10	±	0.03	ab	0.09	±	0.01	bc
			ECM	0.13	±	0.03	ac	0.08	±	0.02	b	0.09	±	0.02	bc	0.09	±	0.02	bc
Cinnamic acid derivative 1	3.8	320	c	0.42	±	0.09	a	0.36	±	0.12	a	0.36	±	0.04	a	0.42	±	0.17	a
			ECM	0.55	±	0.21	a	0.32	±	0.11	a	0.35	±	0.15	a	0.36	±	0.08	a
Cinnamic acid derivative 2	5.2	320	c	0.49	±	0.18	a	0.58	±	0.17	a	0.52	±	0.10	a	0.49	±	0.26	a
			ECM	0.85	±	0.61	a	0.51	±	0.17	a	0.49	±	0.11	a	0.54	±	0.08	a
Cinnamic acid derivative 3	11.5	320	c	0.65	±	0.06	a	0.38	±	0.11	b	0.50	±	0.10	ab	0.50	±	0.06	ab
			ECM	0.66	±	0.05	a	0.46	±	0.13	ab	0.54	±	0.13	ab	0.41	±	0.05	b
Cinnamic acid derivative 4	14.7	320	c	0.22	±	0.05	a	0.10	±	0.03	b	0.13	±	0.03	ab	0.13	±	0.02	ab
			ECM	0.22	±	0.06	a	0.09	±	0.02	b	0.10	±	0.05	b	0.11	±	0.02	b
DHPPG	3.1	320	c	0.45	±	0.15	a	0.74	±	0.22	a	0.62	±	0.13	a	0.81	±	0.41	a
			ECM	0.67	±	0.29	a	0.47	±	0.20	a	0.52	±	0.11	a	0.81	±	0.32	a
Dicoumaroyl-astragalin	39.6	320	c	0.56	±	0.28	a	0.56	±	0.26	a	0.47	±	0.24	a	0.89	±	0.79	a
			ECM	0.81	±	0.56	a	0.45	±	0.35	a	0.71	±	0.23	a	0.89	±	0.38	a
Gallocatechin	2.7	220	c	0.18	±	0.17	a	0.10	±	0.13	a	0.10	±	0.15	a	0.28	±	0.43	a
			ECM	0.31	±	0.29	a	0.08	±	0.11	a	0.12	±	0.19	a	0.31	±	0.30	a
Hyperin	17.8	320	c	0.25	±	0.13	a	0.73	±	0.54	a	0.50	±	0.23	a	0.43	±	0.13	a
			ECM	0.32	±	0.17	a	0.38	±	0.31	a	0.26	±	0.17	a	0.48	±	0.28	a
Kaempherol 3-acetyl-glucoside	30.4	320	c	0.41	±	0.09	ab	0.28	±	0.10	a	0.36	±	0.08	ab	0.28	±	0.11	a
			ECM	0.40	±	0.10	ab	0.30	±	0.11	ab	0.46	±	0.10	b	0.26	±	0.02	a
Kaempherol 3-rhamnoside	24.1	320	c	0.44	±	0.05	a	0.42	±	0.14	a	0.54	±	0.12	a	0.40	±	0.09	a
			ECM	0.43	±	0.08	a	0.43	±	0.12	a	0.52	±	0.07	a	0.43	±	0.05	a
Myricetin 3-acetyl-glucoside	21.1	320	c	7.00	±	2.00	a	6.64	±	2.00	a	6.87	±	0.50	a	7.24	±	2.56	a
			ECM	7.74	±	2.23	a	5.70	±	2.23	a	5.94	±	1.47	a	6.35	±	1.03	a
Myricetin 3-arabifuranoside	16.6	320	c	0.07	±	0.04	a	0.14	±	0.05	a	0.12	±	0.06	a	0.10	±	0.07	a
			ECM	0.08	±	0.05	a	0.09	±	0.06	a	0.06	±	0.06	a	0.11	±	0.10	a
Myricetin 3-arabinose	15.8	320	c	0.03	±	0.06	a	0.08	±	0.06	a	0.06	±	0.07	a	0.05	±	0.06	a
			ECM	0.07	±	0.04	a	0.04	±	0.07	a	0.00	±	0.00	a	0.03	±	0.06	a
Myricetin 3-galactoside	15.3	320	c	0.67	±	0.29	a	1.03	±	0.42	a	0.83	±	0.16	a	0.71	±	0.29	a
			ECM	0.79	±	0.27	a	0.66	±	0.31	a	0.57	±	0.21	a	0.79	±	0.27	a
Myricetin 3-glucoside	15.4	320	c	0.36	±	0.14	a	0.50	±	0.19	a	0.41	±	0.10	a	0.43	±	0.17	a
Myricetin 3-glucuronide			ECM	0.46	±	0.16	a	0.31	±	0.17	a	0.27	±	0.10	a	0.42	±	0.18	a
Myricetin 3-rhamnoside	17.4	320	c	10.68	±	1.30	a	14.43	±	3.82	a	13.51	±	1.49	a	13.13	±	3.25	a
			ECM	10.89	±	2.16	a	11.60	±	5.4	a	10.51	±	2.77	a	12.56	±	3.13	a
p-OH-cinnamic acid derivative	28.7	320	c	0.11	±	0.04	a	0.08	±	0.02	b	0.08	±	0.01	ab	0.09	±	0.03	ab
			ECM	0.09	±	0.02	ab	0.07	±	0.02	ab	0.10	±	0.03	a	0.07	±	0.02	ab

p-OH-cinnamic acid glucoside	10.3	320	c	3.11 ± 0.45 b	1.79 ± 0.48 a	2.39 ± 0.31 ab	0.58 ± 0.72 ab
			ECM	3.01 ± 0.36 bc	2.15 ± 0.69 ac	2.61 ± 0.30 bc	2.35 ± 0.45 ab
Quercetin 3-acetyl-glucoside	26.2	320	c	4.52 ± 1.32 a	4.46 ± 1.57 a	4.46 ± 1.71 a	6.05 ± 1.33 a
			ECM	5.60 ± 1.27 a	4.74 ± 1.51 a	6.11 ± 0.90 a	6.11 ± 1.27 a
Quercetin 3-arabinoside	20.4	320	c	0.28 ± 0.12 ab	0.35 ± 0.11 ab	0.29 ± 0.06 ab	0.35 ± 0.12 ab
			ECM	0.26 ± 0.08 ab	0.29 ± 0.12 ab	0.18 ± 0.07 a	0.38 ± 0.08 b
Quercetin 3-glucoside	18.3	320	c	0.14 ± 0.08 a	0.23 ± 0.12 a	0.18 ± 0.10 a	0.22 ± 0.08 a
Quercetin 3-glucuronide			ECM	0.21 ± 0.07 a	0.11 ± 0.11 a	0.11 ± 0.10 a	0.23 ± 0.08 a

Stems				Clone	Lines		
	RT	nm	T	A	23	44	65
Apigenin derivative	39.7	320	c	0.04 ± 0.03 a	0.06 ± 0.02 a	0.06 ± 0.03 a	0.09 ± 0.04 a
			ECM	0.06 ± 0.02 a	0.07 ± 0.02 a	0.07 ± 0.03 a	0.06 ± 0.03 a
Betuloside	9.5	220	c	7.08 ± 1.60 a	7.07 ± 0.77 a	7.23 ± 0.95 a	7.27 ± 1.93 a
			ECM	6.30 ± 0.68 a	7.07 ± 0.67 a	6.52 ± 2.32 a	6.97 ± 0.45 a
(+)-Catechin	7.4	220	c	9.23 ± 1.22 ab	6.85 ± 0.96 b	6.17 ± 0.96 b	8.21 ± 1.82 ab
			ECM	9.93 ± 1.73 a	7.77 ± 1.48 ab	6.70 ± 1.46 b	6.98 ± 0.98 ab
Catechin derivative	1.5	220	c	0.16 ± 0.03 a	0.14 ± 0.05 a	0.13 ± 0.02 a	0.17 ± 0.04 a
			ECM	0.15 ± 0.05 a	0.12 ± 0.02 a	0.13 ± 0.01 a	0.13 ± 0.07 a
Catechin xyloside	6.5	220	c	0.47 ± 0.11 a	0.46 ± 0.06 a	0.36 ± 0.13 a	0.54 ± 0.17 a
			ECM	0.37 ± 0.14 a	0.43 ± 0.13 a	0.30 ± 0.12 a	0.58 ± 0.09 a
Cinnamic acid derivative 1	3.7	320	c	0.11 ± 0.05 a	0.09 ± 0.02 a	0.08 ± 0.02 a	0.10 ± 0.02 a
			ECM	0.15 ± 0.05 a	0.10 ± 0.02 a	0.11 ± 0.02 a	0.07 ± 0.04 a
Cinnamic acid derivative 2	5.0	320	c	0.13 ± 0.05 a	0.14 ± 0.03 a	0.11 ± 0.03 a	0.18 ± 0.03 a
			ECM	0.17 ± 0.07 a	0.14 ± 0.03 a	0.13 ± 0.04 a	0.16 ± 0.08 a
DHPPG	3.0	220	c	1.46 ± 0.64 a	2.42 ± 1.13 ab	2.20 ± 0.80 ab	3.92 ± 0.75 b
			ECM	1.61 ± 0.36 a	2.91 ± 0.86 b	2.93 ± 1.91 ab	3.38 ± 1.41 ab
Dicoumaroyl-astragalin	39.6	320	c	0.50 ± 0.34 a	0.46 ± 0.22 a	0.46 ± 0.26 a	0.81 ± 0.23 a
			ECM	0.50 ± 0.20 a	0.52 ± 0.16 a	0.58 ± 0.14 a	0.51 ± 0.25 a
Gallocatechin	2.7	220	c	1.12 ± 0.25 a	1.92 ± 0.36 ab	1.58 ± 0.33 ab	2.02 ± 0.34 b
			ECM	1.16 ± 0.21 a	1.97 ± 0.43 b	1.63 ± 0.36 ab	1.66 ± 0.32 ab
Isorhamnetin 3-glucoside	21.4	320	c	0.20 ± 0.11 a	0.22 ± 0.13 a	0.17 ± 0.12 a	0.32 ± 0.20 a
			ECM	0.23 ± 0.12 a	0.16 ± 0.03 a	0.15 ± 0.12 a	0.14 ± 0.09 a
Kaempherol 3-rhamnoside	24.0	320	c	0.12 ± 0.08 a	0.15 ± 0.04 a	0.11 ± 0.03 a	0.19 ± 0.05 a
			ECM	0.15 ± 0.08 a	0.12 ± 0.08 a	0.13 ± 0.05 a	0.15 ± 0.11 a
Luteolin derivative	21.1	320	c	0.03 ± 0.05 a	0.04 ± 0.08 a	0.03 ± 0.07 a	0.07 ± 0.07 a
			ECM	0.03 ± 0.05 a	0.01 ± 0.02 a	0.04 ± 0.06 a	0.03 ± 0.06 a
p-OH-cinnamic acid glucoside	10.3	320	c	2.47 ± 0.70 b	1.14 ± 0.23 a	1.15 ± 0.24 a	1.38 ± 0.32 ac
			ECM	2.01 ± 0.40 bc	1.22 ± 0.16 a	1.03 ± 0.28 a	0.95 ± 0.16 a
Platyfylloside	20.0	220	c	7.76 ± 1.85 a	9.48 ± 1.55 a	8.37 ± 1.66 a	10.91 ± 3.08 a
			ECM	6.69 ± 2.31 a	9.60 ± 2.57 a	7.79 ± 2.81 a	6.52 ± 4.92 a
Salidroside	2.2	220	c	0.56 ± 0.15 a	0.51 ± 0.15 a	0.38 ± 0.14 a	0.57 ± 0.20 a
			ECM	0.79 ± 0.33 a	0.42 ± 0.11 a	0.39 ± 0.14 a	0.50 ± 0.18 a

Roots				Clone		Lines					
	RT	nm	T	A		23		44		65	
(+)-Catechin	7.3	220	c	10.75 ± 0.44	a	8.84 ± 1.96	a	8.06 ± 1.90	a	7.70 ± 2.31	a
			ECM	10.9 ± 2.79	a	11.6 ± 2.52	a	7.42 ± 2.80	a	6.59 ± 1.16	a
Catechin derivative	1.5	220	c	0.07 ± 00.01	a	0.10 ± 0.01	a	0.09 ± 0.02	a	0.09 ± 0.02	a
			ECM	0.08 ± 0.01	a	0.11 ± 0.01	a	0.09 ± 0.04	a	0.08 ± 0.01	a
Cinnamic acid derivative	10.2	320	c	0.71 ± 0.33	a	0.17 ± 0.05	a	0.17 ± 0.10	a	0.37 ± 0.23	a
			ECM	0.53 ± 0.30	a	0.19 ± 0.02	a	0.20 ± 0.13	a	0.24 ± 0.14	a
Condensed tannin precursor 1	8.6	220	c	13.45 ± 3.64	a	12.66 ± 3.20	a	10.95 ± 2.75	a	10.48 ± 1.04	a
			ECM	13.73 ± 3.84	a	17.90 ± 5.42	a	7.13 ± 2.79	a	13.73 ± 1.05	a
Condensed tannin precursor 2	10.0	220	c	5.61 ± 0.29	a	5.07 ± 0.78	a	4.73 ± 0.81	a	5.49 ± 1.55	a
			ECM	4.48 ± 1.24	a	5.13 ± 0.96	a	4.81 ± 1.60	a	5.06 ± 0.38	a
Condensed tannin precursor 3	10.5	220	c	9.66 ± 1.44	ab	8.50 ± 1.83	ab	8.17 ± 1.58	ab	8.02 ± 1.40	ab
			ECM	11.48 ± 3.05	a	11.26 ± 2.71	a	5.66 ± 1.97	b	7.06 ± 0.66	ab
Condensed tannin precursor 4	11.1	220	c	6.36 ± 1.12	ab	5.21 ± 1.60	ab	4.75 ± 1.32	ab	4.51 ± 0.39	ab
			ECM	7.51 ± 2.37	a	7.63 ± 2.30	a	2.78 ± 1.13	b	3.86 ± 0.61	ab
Dicatechin	6.5	220	c	0.48 ± 0.25	a	0.44 ± 0.11	a	0.26 ± 0.04	a	0.38 ± 0.06	a
			ECM	0.29 ± 0.10	a	0.26 ± 0.10	a	0.26 ± 0.08	a	0.51 ± 0.13	a
Ellagic acid	18.5	320	c	0.00 ± 0.00	a	0.19 ± 0.18	a	0.15 ± 002	a	0.00 ± 0.00	a
			ECM	0.00 ± 0.00	a	0.23 ± 0.08	a	0.12 ± 0.07	a	0.00 ± 0.00	a
Ellagic acid derivative	17.7	320	c	0.00 ± 0.00	a	0.07 ± 0.08	a	0.06 ± 0.09	a	0.06 ± 0.04	a
			ECM	0.04 ± 0.07	a	0.06 ± 0.07	a	0.05 ± 0.05	a	0.09 ± 0.12	a
Monogalloyl glucose	0.7	220	c	0.06 ± 0.01	a	0.06 ± 0.01	a	0.07 ± 0.01	a	0.06 ± 0.02	a
			ECM	0.07 ± 0.02	a	0.07 ± 0.02	a	0.07 ± 0.03	a	0.06 ± 0.01	a
Methylluteolin 7-glucoside	21.3	320	c	0.09 ± 0.18	a	0.07 ± 0.09	a	0.00 ± 0.00	a	00.09 ± 0.07	a
			ECM	0.00 ± 0.00	a	0.07 ± 0.08	a	0.11 ± 0.20	a	0.03 ± 0.06	a
Platyfylloside	19.9	220	c	0.01 ± 0.02	a	0.24 ± 0.20	a	0.12 ± 0.07	a	0.00 ± 0.00	a
			ECM	0.00 ± 0.00	a	0.27 ± 0.20	a	0.16 ± 0.08	a	0.00 ± 0.00	a
