# Supplementary material for: Phenolic compounds in ectomycorrhizal interaction of lignin modified silver birch
Source: BMC Plant Biol. 2009 Sep 29;9:124. doi: 10.1186/1471-2229-9-124 (PMC2763875; doi:10.1186/1471-2229-9-124)
Supplement: Additional file 2 — Alignment of predicted amino acid sequence of putative silver birch PP2A. Alignment of the partial silver birch protein phosphatase 2A regulatory subunit (BpPP2A) amino acid sequence with the PP2Asequences of Medicago sativa subsp. x varia [GenBank: [AAG29593]], Arabidopsis thaliana [GenBank: [NP_172790]], Zea mays [GenBank: [NP_001105839]] and Oryza sativa [EMBL: [CAB51803]]. Conserved amino acids present in all sequences are highlighted in indigo blue and similar with blue-grey. [file 1471-2229-9-124-S2.PDF]

```
                      *        20         *        40         *
B. pendula   --------------------------------------------------
M. sativa    --MADEPLYPIAVLIDELKNDDIQLRLNSIRRLSTIARALGEERTRKELI
A. thaliana  MSMVDEPLYPIAVLIDELKNDDIQRRLNSIKRLSIIARALGEERTRKELI
Z. mays      MAMIDEPLYPIAVLIDELKNEDIQLRLNSIRRLSTIARALGEERTRKELI
O. sativa    MASMDEPLYPIAILIDELKNEDIQLRLNSIRRLSTIARALGEERTRKELI

                    60         *        80         *       100
B. pendula   --------------------------YVGGVEHAHVLLPPLETLCTVEET
M. sativa    PFLTENNDDDDEVLLAMAEELGVFIPYVGGVEHASVLLPPLEAFCSVEET
A. thaliana  PFLSENNDDDDEVLLAMAEELGGFILYVGGVEYAYVLLPPLETLSTVEET
Z. mays      PFLSENNDDEDEVLLAMAEELGVFIPYVGGVEHAHVLLPPLETLCTVEET
O. sativa    PFLSENNDDEDEVLLAMAEELGVFIPYVGGVEHAHVLLPPLETLCTVEET

                      *       120         *       140         *
B. pendula   CVREKALESLCRIGSQMRESDLVDWFIPLVKRLAAGEWFTARVSACGLFH
M. sativa    CVRDKAVESLCRIGSQMRESDLVEYFIPLVKRLAAGEWFTARVSACGLFH
A. thaliana  CVREKAVDSLCRIGAQMRESDLVEHFTPLAKRLSAGEWFTARVSACGIFH
Z. mays      CVRDKAVESLCRIGAQMKETDIVDWFIPVVKRLAAGEWFTARVSSCGLFH
O. sativa    CVRDKAVESLCRIGAQMKESDIVDWFVPVVKRLAAGEWFTARVSSCGLFH

                   160         *       180         *       200
B. pendula   IAYPSAPEMLKIELRSIYSQLCQDDMPMVRRSAATNLGKFAATVESAHLK
M. sativa    IAYPSAPEASKTELRSIYSQLCQDDMPMVRRSAATNLGKFAATVEYTHLK
A. thaliana  IAYPSAPDVLKTELRSIYGQLCQDDMPMVRRAAATNLGKFAATIESAHLK
Z. mays      IAYPSASDQLKTELRTIYGQLCQDDMPMVRRAAASNLGKFAATVEQNHLK
O. sativa    IAYPSAPDQLKAELRTIYGQLCQDDMPMVRRAAASNLGKFAATVEQNYLK

                      *       220         *       240         *
B. pendula   AEIMSMFEDLTQDDQDSVRLLAVEGCAALGKLLEPQDCVAHILPVIVSFS
M. sativa    ADIMSIFDDLTQDDQDSVRLLAVEGCAALGKLLEPQDCVAHILPVIVNFS
A. thaliana  TDIMSMFEDLTQDDQDSVRLLAVEGCAALGKLLEPQDCVAHILPVIVNFS
Z. mays      TEIMSIFDDLTQDDQDSVRLLAVEGCAALGKLLEPQDCVAHILPVIVNFS
O. sativa    TEVMSIFDDLTQDDQDSVRLLAVEGCAALGKLLEPQDCVAHILPVIVNFS

                   260         *       280         *       300
B. pendula   QDKSWRVRYMVANQLYELCEAVGPEPTRTELVPAYVRLLRDNESEVRIAA
M. sativa    QDKSWRVRYMVANQLYELCEAVGPEPTRAELVPAYVRLLRDNEAEVRIAA
A. thaliana  QDKSWRVRYMVANQLYELCEAVGPEPTRTDLVPAYARLLCDNEAEVRIAA
Z. mays      QDKSWRVRYMVANQLYELCEAVGPEPTRADLVPAYVRLLRDNEAEVRIAA
O. sativa    QDKSWRVRYMVANQLYELCEAVGPEHSREQLVPAYVRLLRDNEAEVRIAA

                      *       320         *       340         *
B. pendula   AGKVTKFCRILNPELAIQHILPCVKELSSDSSQHVRSALASVIMGMAPVL
M. sativa    AGKVTKFCRILSPDLAIQHILPCVKELSSDSSQHVRSALASVIMGMAPVL
A. thaliana  AGKVTKFCRILNPELAIQHILPCVKELSSDSSQHVRSALASVIMGMAPVL
Z. mays      AGKVTKFCRILNPQLSVQHILPCVKELSSDSSQHVRSALASVIMGMAPVL
O. sativa    AGKVTKFCRILSPQLAIQHILPCVKELSSDSSQHVRSALASVIMGMAPVL

                   360         *       380         *       400
B. pendula   GKDATIEQLLPIFLSLLKDEFPDVRLNIISKLDQVNQVIGIDLLSQSLLP
M. sativa    GKEATIEQLLHIFLSLLKDEFPDVRINIISKLDQVNQVIGIDLLSQSLLP
A. thaliana  GKDATIEHLLPIFLSLLKDEFPDVRLNIISKLDQVNQVIGIDLLSQSLLP
Z. mays      GKDATIEQLVPIFLSLLKDEFPDVRLNIISKLDQVNQVIGIDLLSQSLLP
O. sativa    GKEATIEQLLPIFLSLLKDEFPDVRLNIISKLDQVNQVIGIDLLSQSLLP

                      *       420         *       440         *
B. pendula   AIVELAEDRHWRVRLAIIEYIPLLASQLGVGFFDDKLGALCMQWLKDKVY
M. sativa    AIVELAEDRHWRVRLAIIEYIPLLASQLGVGFFDDKLGALCMQWLQDKVH
A. thaliana  AIVELAEDRHWRVRLAIIEYIPLLASQLGVGFFDEKLGALCMQWLQDKVH
Z. mays      AIVELAEDRHWRVRLAIIEYIPLLASQLGVGFFDDKLGALCMQWLEDKVF
O. sativa    AIVELAEDRHWRVRLAIIEYIPLLASQLGVGFFDDKLGALCMQWLEDKVF

                   460         *       480         *       500
B. pendula   SIRDAAANNLKRLAEEFGPEWAMQHIIPQVLEMVNNQHYLYRMTILRAIS
M. sativa    SIREAAANNLKRLAEEFGPEWAMQHIIPQVLEMITNPHYLYRMTILRAIS
A. thaliana  SIREAAANNLKRLAEEFGPEWAMQHIVPQVLEMINNPHYLYRMTILRAVS
Z. mays      SIRDAAANNLKRLAEEFGPEWAMQHIIPQVLEKINNPHYLYRMTILQAIS
O. sativa    SIRDAAANNLKRLAEEFGPEWAMQHIIPQVLEKINNPHYLYRMTILQAIS

                      *       520         *       540         *
B. pendula   LLAPVMGSEITNSNLLPVVINASKDRVPNIKFNEAKVFQSLIPIVDHSVV
M. sativa    LLAPVMGSEVTCSKLLPAVVAASKDRVPNIKFNVAKVLESIFPIVDQSVV
A. thaliana  LLAPVMGSEITCSKLLPAVITASKDRVPNIKFNVAKMMQSLIPIVDQAVV
Z. mays      LLAPVMGAEITCQKLLPVVINSSKDRVPNIKFNVAKVLQSLVPILDQSTV
O. sativa    LLAPVMGAEITCQQLLPVVINSSKDRVPNIKFNVAKVLQALIPILDQSVV

                   560         *       580
B. pendula   EKTIRPCLVELGEDPDVDVRFFANQALESIDHVMMSS
M. sativa    EKTIRPCLVELGEDPDVDVRFFSNQALQAIDHVMMSS
A. thaliana  ENMIRPCLVELSEDPDVDVRYFANQALQSIDNVMMSS
Z. mays      KPCL----VELSEDPDVDVRYYANQALQACDQMMVSS
O. sativa    EKNVKPCLVELSEDPDVDVRYYANQALQACDQIMMSS
```
